# Prevalence and characteristics of participants in *Dry January* 2024: findings from a general population survey in France

**DOI:** 10.3389/fpubh.2024.1466739

**Published:** 2024-12-02

**Authors:** Louis-Ferdinand Lespine, Diane François, Julie Haesebaert, Jean-Michel Delile, Myriam Savy, Benjamin Tubiana-Rey, Mickael Naassila, Julia de Ternay, Benjamin Rolland

**Affiliations:** ^1^Service Universitaire d’Addictologie de Lyon (SUAL), Le Vinatier Psychiatrie Universitaire Lyon Métropole, Bron, France; ^2^Direction de la Recherche Clinique et de l’Innovation (DRCI), Le Vinatier Psychiatrie Universitaire Lyon Métropole, Bron, France; ^3^Research on Healthcare Performance (RESHAPE), INSERM U1290, Hospices Civils de Lyon, Lyon, France; ^4^Fédération Addiction, Paris, France; ^5^Association Addictions France, Paris, France; ^6^Groupe de Recherche sur l’Alcool et les Pharmacodépendances (GRAP), INSERM U1247, Université de Picardie Jules Verne, Amiens, France; ^7^Service Universitaire d’Addictologie de Lyon (SUAL), Hôpital Edouard Herriot, Hospices Civils de Lyon, Lyon, France; ^8^Université Claude Bernard Lyon 1, CNRS, INSERM, Centre de Recherche en Neurosciences de Lyon CRNL U1028 UMR5292, PSYR2, Bron, France

**Keywords:** Dry January, alcohol, temporary abstinence campaign, prevalence, participation

## Abstract

**Background:**

Dry January is a one-month alcohol abstinence challenge for the general population running since 2013 in the United Kingdom, and 2020 in France. Dry January has gained increasing popularity among the public, but studies assessing the individual characteristics associated with awareness and participation remain sparse.

**Methods:**

Using quota sampling, a representative sample of 5,000 French adults completed an online cross-sectional survey between 8 and 17th January 2024. Chi-square tests and binary logistic regressions were used to explore demographic and alcohol-related characteristics associated with awareness of the Dry January campaign as well as participation.

**Results:**

Among 4,075 past-year alcohol users, 2,468 (61%) were aware of the “Dry January” campaign, of whom 497 (20%) were participants (12% of all alcohol users). Extrapolated to the entire adult French population, this corresponds to an estimated 4.5 million people participating in the Dry January 2024. Awareness was comparable between genders and across age groups, but was greater among individuals with higher occupational status, and lower among those living in Eastern regions of France. Individuals aware of the campaign were more likely to self-evaluate their drinking as “at risk” and to report high-risk consumption. Participation rates did not differ by gender, occupational status, or region but decreased with age. Compared to non-participants, Dry January participants were more likely to self-identify their drinking as at-risk, to be concerned about health-related effects of alcohol, to be concerned about their control (or lack thereof) over drinking, and to report hazardous use or possible alcohol use disorder. However, no evidence was found for an association between high-risk consumption based on AUDIT-C and participation. Among participants, aiming for reduction (vs. abstinence) and official registration (vs. unofficial participation), were associated with worsened alcohol-related measures.

**Conclusion:**

This study indicates a stable level of awareness, but encouraging participation in Dry January in France. The results also confirm that temporary alcohol abstinence campaigns primarily attract high-risk drinkers and individuals reporting harmful consequences related to alcohol.

## 1 Introduction

Alcohol consumption is a leading risk factor for premature mortality, morbidity and social harm (1), and a growing body of evidence indicates that there is no safe level of alcohol consumption (2, 3). However, alcohol consumption remains ingrained in the cultural habits of many countries, particularly within Europe, the Americas, and the Western Pacific Region (1). In France, a national survey conducted in 2021 indicated that 85% of adult population had consumed alcohol in the past year, with 39 and 8% reporting weekly and daily consumption, respectively (4). Moreover, 54% indicated that they had consumed alcohol in the past week, and 22% exceeded low-risk drinking guidelines (5). These findings call for continued efforts to reduce alcohol consumption within the general population.

In this context, temporary alcohol abstinence campaigns (TAACs), which challenge the general population to abstain from alcohol during a fixed period (typically 1 month), might represent promising and cost-effective initiatives to promote behavioral changes and improve general health. In addition to the potential benefits for health and well-being, TAACs provide an opportunity for alcohol users to question their relationship with alcohol, as well as the social and cultural norms associated with drinking. As they do not necessarily promote long-term abstinence, TAACs could be conceptualized as harm reduction programs applied to the general population (6). Importantly, TAACs have spread over the years, with campaigns now established in many countries, including the United Kingdom (UK) (7), France (8), Switzerland (9), Iceland (10), the Netherlands (11), the United States (12), Belgium (13), Canada (14), Hungary (15) or Australia (16).

Prospective studies have shown that TAAC participation is associated with global benefits lasting up to 6–8 months, including less frequent drinking, lower volumes of alcohol consumption, increased confidence in resisting alcohol (but see (17)), a decrease in Alcohol Use Disorders Identification Test (AUDIT) scores, reduced automaticity and craving, and improvements in mental and physical health (6, 17–22). However, one study reported that increased participation in Dry January—a TAAC launched in UK in 2013 by Alcohol Change UK (formerly Alcohol Concern)—, was not associated with significant declines in alcohol consumption at the population level in England (23). In contrast, a recent study conducted in Thailand on the TAAC Buddhist Lent period (3 months) showed a decrease in *per capita* alcohol consumption before and after the campaign (24). Regarding participants’ profile, research has indicated that women and individuals with a higher socio-economic status are more likely to take part in TAACs (6, 25). Compared to non-participating drinkers, TAAC participants were also found to be more likely to classify themselves as heavier drinkers, to be concerned about the health consequences of drinking and their control (or lack thereof) over drinking, and to be higher risk drinkers (6). However, these observations are primarily drawn from cohort studies, where TAAC participants were mostly “registrants,” i.e., people who sign up to participate “officially” and to receive supportive emails and tips. It has been suggested that registrants are the most concerned about their drinking and/or are already in the “planning” or “action” phases of behavior change (20, 26, 27).

Although anecdotal evidence, gray literature and media coverage suggest a growing enthusiasm for TAACs, scientific literature exploring awareness and participation among the general population remains sparse (6). This is crucial for optimizing communication strategies and ensuring continued success of such campaigns. As part of a research project called JANOVER, which aims to provide insights into participation in Dry January in France (“le Défi de Janvier”), this online cross-sectional study aimed to estimate the prevalence of awareness and participation, as well as their associated demographic and drinking characteristics. We also assessed the registration status among Dry January participants and their goals, i.e., abstinence or reduction—two important but overlooked outcomes so far.

## 2 Materials and methods

### 2.1 Design

An online cross-sectional survey was conducted between 8^th^ and 17^th^ January 2024. The study sample was recruited from an online panel managed by the French market survey company, Selvitys®. The panel consisted of contact details for members of the public, aged 18 years and older, living in France, who had expressed an interest in participating in research surveys. Quota sampling was used based on age, gender, occupational status, and region of residence to ensure that the sample was broadly representative of the French general population. The protocol was reviewed and approved by the ethics committee of Le Vinatier Hospital (CEREVI/2023/012).

### 2.2 Measures

Demographics included gender, age, region, and occupational status, as defined by the National Institute of Statistics and Economic Studies (NI-SES). Since previous studies suggested that individuals with a high socioeconomic status were more likely to take part in TAACs (6, 25), the NI-SES classification was recoded into a dichotomous variable indicating whether respondents had a high occupational status (e.g., executive and higher intellectual profession).

Alcohol use was assessed using the 10-item Alcohol Use Disorders Identification Test (AUDIT (28)) with total scores ranging from 0 to 40, categorized as low risk (<8), hazardous use (8 to 15) and harmful use or possible dependence (>15) (29).

Alcohol misuse (or high-risk drinking) was further defined as scoring ≥5 on the AUDIT-Consumption subscale (AUDIT-C; items 1–3 of the AUDIT) (30). This cut-off was considered appropriate for this study, as it identified 23% high-risk drinkers (928/4,075), corresponding to the proportion of adults exceeding low-risk drinking guidelines in previous national studies (5).

Although self-reported (which is not standard practice), Alcohol Use Disorder (AUD) was investigated using the 11 criteria from the Diagnostic and Statistical Manual of Mental Disorders 5^th^ edition (DSM-5) for substance use disorder (31), categorized as none (<2 criteria), mild (2 or 3 criteria), moderate (4 or 5 criteria), or severe (≥6 criteria).

Respondents also indicated their level of concern about the effect of their drinking on their health and about their control, or lack thereof, over their drinking, using 10-point scales (anchored by “not at all*”* and “extremely”) (21). The scores were recoded into a dichotomous variable indicating low (from 1 to 5) or high (≥6) concern.

At-risk drinking recognition was evaluated by asking respondents whether they felt “like they were drinking more than they should” (32).

Finally, respondents were asked whether they were aware of the campaign called “Dry January.” Those who responded positively were asked whether they were currently participating. Participants in Dry January were asked about their goal (i.e., abstinence or reduction) and whether they had registered for “official” participation.

### 2.3 Data analysis

Descriptive statistics are presented as the number and percentage (n, %). We compared demographic and alcohol-related characteristics across the following groups: (i) individuals who were aware of Dry January vs. those who were not, (ii) participants in Dry January vs. non-participants, (iii) participants who aimed for reduction vs. those who aimed for abstinence, and (iv) participants who registered vs. those who did not. Comparisons were performed using the Chi-squared test or Fisher’s exact test. Logistic regressions were used to further analyze the associations between demographic and alcohol measures, and the four binary outcomes: awareness of the campaign (0 = no, 1 = yes), participation (0 = no, 1 = yes), registration (0 = no, 1 = yes), and goal (0 = abstinence, 1 = reduction). Awareness and participation were considered primary outcomes, while registration and goal were considered secondary outcomes. To avoid multicollinearity, alcohol-related measures were analyzed individually while controlling for demographic factors. Results are reported as adjusted odds ratio (aOR) and 95% confidence intervals (95% CI). All statistical analyses were performed using the JASP software,^1^ the level of significance set at 0.05.

## 3 Results

### 3.1 Prevalence of awareness and participation in Dry January 2024

Of the 5,000 respondents, 4,075 (82%) had consumed alcohol in the past year. Among them, 2,468 (61%) were aware of the Dry January campaign, with 497 indicating they were currently participating in the challenge, representing 12% of past-year alcohol users and 20% of those aware of the campaign.

### 3.2 Profile of drinkers aware of Dry January

**Figure 1:**
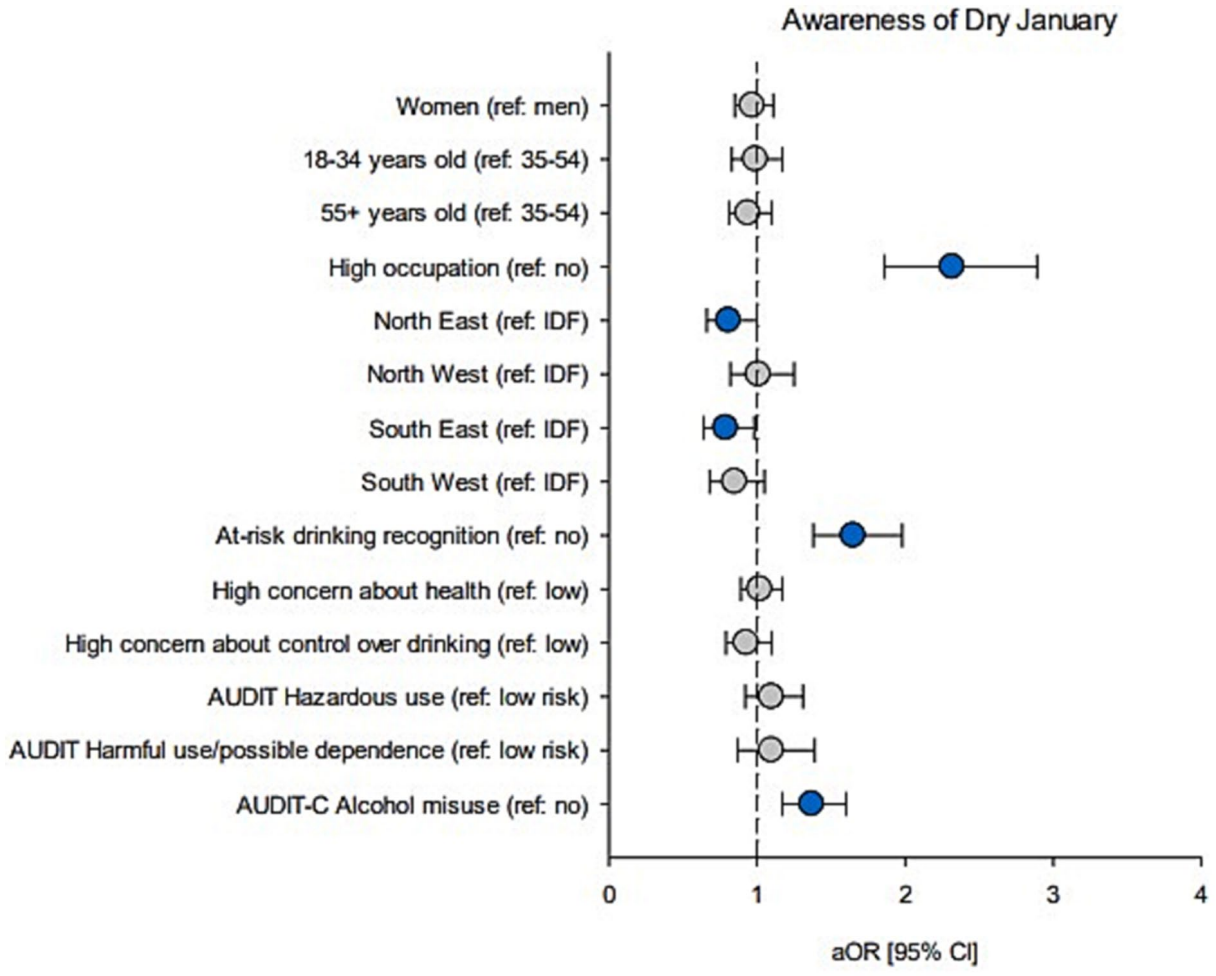
Associations between demographics and alcohol measures, and awareness of Dry January. Forest plot shows adjusted OR (circles) and 95% CI (bars); adjusted for demographics. Blue circles: significant estimates (*p* < 0.05).

Results are reported in Table 1 (demographics), Table 2 (drinking characteristics) and Table 3 (aOR and 95% CI). Figure 1 also reports aOR and 95% CI. Among past-year drinkers, awareness was comparable between men and women (61% vs. 60% respectively) and across age groups (61% among 18–34-year-olds, 62% in 35–54-year-olds, and 59% among people aged 55+). However, individuals with higher occupational status were more likely to be aware of the campaign (77% vs. 58%, aOR = 2.32). Awareness also differed across regions, ranging from 58% in South and North East to 65% in Ile-de-France (i.e., the Paris region). Logistic regression confirmed that living in Eastern regions was significantly associated with a lower likelihood of being aware of Dry January compared to the Paris region (aORs = 0.81 and 0.79).

**Table 1:** Awareness, participation, a goal of reduction (rather than abstinence), and registration, by demographics.

		Among past-year drinkers	Among people aware of the campaign	Among participants	
	Past-year drinkers *N* = 4,075	Aware of the campaign *n* = 2,468 (61%)	Participants *n* = 497 (20%)	Aim for reduction *n* = 95 (19%)	Registration *n* = 102 (21%)
Gender, n (%)		χ^2^_(1)_ = 0.741 (*p* = 0.389)	χ^2^_(1)_ = 1.633 (*p* = 0.201)	χ^2^_(1)_ = 1.369 (*p* = 0.242)	χ^2^_(1)_ = 0.027 (*p* = 0.869)
Men	*n* = 2,050	1,255 (61%)	240 (19%)	51 (21%)	50 (21%)
Women	*n* = 2,025	1,213 (60%)	257 (21%)	44 (17%)	52 (20%)
Age, n (%)		χ^2^_(2)_ = 3.795 (*p* = 0.150)	**χ**^**2**^_**(2)**_ **= 51.053 (*p* < 0.001)**	χ^2^_(2)_ = 1.612 (*p* = 0.447)	**χ**^**2**^_**(2)**_ **= 14.305 (*p* < 0.001)**
18–34	*n* = 959	584 (61%)	172 (29%)	38 (22%)	51 (30%)
35–54	*n* = 1,384	863 (62%)	176 (20%)	32 (18%)	31 (18%)
55+	*n* = 1,732	1,021 (59%)	149 (15%)	25 (17%)	20 (13%)
High occupational status, n (%)		**χ**^**2**^_**(1)**_ **= 65.77 (*p* < 0.001)**	χ^2^_(1)_ = 0.874 (*p* = 0.350)	χ^2^_(1)_ = 2.599 (*p* = 0.107)	χ^2^_(1)_ = 0.294 (*p* = 0.588)
Yes	*n* = 518	398 (77%)	87 (22%)	22 (25%)	16 (18%)
No	*n* = 3,557	2,070 (58%)	410 (20%)	73 (18%)	86 (21%)
Region, n (%)		**χ**^**2**^_**(4)**_ **= 13.46 (*p* = 0.009)**	χ^2^_(4)_ = 8.437 (*p* = 0.077)	χ^2^_(4)_ = 0.608 (*p* = 0.962)	**χ**^**2**^_**(4)**_ **= 17.58 (*p* = 0.001)**
IDF^a^	*n* = 710	461 (65%)	103 (22%)	20 (19%)	34 (33%)
North East	*n* = 952	554 (58%)	128 (23%)	26 (20%)	22 (17%)
North West	*n* = 829	525 (63%)	103 (20%)	17 (17%)	13 (13%)
South East	*n* = 825	478 (58%)	82 (17%)	16 (20%)	21 (26%)
South West	*n* = 759	450 (59%)	81 (18%)	16 (20%)	12 (15%)

a Ile-de-France (the Parisian region).

Bold: statistically significant. NB: % are presented by rows (i.e., by demographic group). For example, among 2,050 male drinkers, 1,255 (61%) were aware of the campaign (2^nd^ column). Among them, 240 (19%) were participants (3^rd^ column).

**Table 2:** Drinking characteristics according to awareness and participation.

		Among past-year drinkers		Among people aware of the campaign	
	Past-year drinkers *n* = 4,075	Unaware *n* = 1,607	Aware *n* = 2,468	Non-participants *n* = 1,971	Participants *n* = 497
At-risk drinking recognition, n (%)		**χ**^**2**^_**(1)**_ **= 36.222 (*p* < 0.001)**		**χ**^**2**^_**(1)**_ **= 52.102 (*p* < 0.001)**	
	705 (17%)	207 (13%)	498 (20%)	340 (17%)	158 (32%)
High concern about health, n (%)		χ^2^_(1)_ = 0.606 (*p* = 0.436)		**χ**^**2**^_**(1)**_ **= 27.326 (*p* < 0.001)**	
	1,243 (31%)	479 (30%)	764 (31%)	562 (29%)	202 (41%)
High concern about (lack of) control, n (%)		χ^2^_(1)_ = 0.069 (*p* = 0.793)		**χ**^**2**^_**(1)**_ **= 39.469 (*p* < 0.001)**	
	735 (18%)	293 (18%)	442 (18%)	305 (15%)	137 (28%)
AUDIT, n (%)		χ^2^_(2)_ = 5.171 (*p* = 0.075)		**χ**^**2**^_**(2)**_ **= 85.898 (*p* < 0.001)**	
Low risk	2,968 (73%)	1,202 (75%)	1,766 (72%)	1,475 (75%)	291 (59%)
Hazardous use	747 (18%)	273 (17%)	474 (19%)	364 (18%)	110 (22%)
Harmful use/possible dependence	360 (9%)	132 (8%)	228 (9%)	132 (7%)	96 (19%)
AUDIT-C: higher risk, n (%)		**χ**^**2**^_**(1)**_ **= 19.628 (*p* < 0.001)**		χ^2^_(1)_ = 0.629 (*p* = 0.428)	
	928 (23%)	308 (19%)	620 (25%)	502 (25%)	118 (24%)

Bold: statistically significant.

**Table 3:** Associations between demographic and alcohol measures, and awareness and participation.

	Awareness		Participation	
	OR [95% CI]^a^	*p*-value	OR [95% CI]^a^	*p*-value
Gender (ref: men)				
Women	0.97 [0.85–1.11]	0.635	0.99 [0.81–1.22]	0.942
Age (ref: 35–54)				
18–34	0.99 [0.83–1.17]	0.893	**1.65 [1.29–2.10]**	**< 0.001**
55+	0.94 [0.81–1.10]	0.446	**0.69 [0.54–0.89]**	**0.004**
High occupational status (ref: no)	**2.32 [1.86–2.89]**	**< 0.001**	1.02 [0.78–1.34]	0.866
Region (ref: IDF)^b^				
North East	**0.81 [0.66–1.00]**	**0.044**	1.02 [0.75–1.38]	0.900
North West	1.01 [0.82–1.25]	0.899	0.87 [0.64–1.19]	0.390
South East	**0.79 [0.64–0.98]**	**0.030**	0.76 [0.55–1.05]	0.096
South West	0.85 [0.68–1.05]	0.130	0.79 [0.57–1.11]	0.171
At-risk drinking recognition (ref: no)	**1.65 [1.38–1.98]**	**< 0.001**	**2.09 [1.66–2.62]**	**< 0.001**
High concern about health (ref: low)	1.02 [0.89–1.17]	0.772	**1.63 [1.32–2.01]**	**< 0.001**
High concern about control (ref: low)	0.93 [0.79–1.10]	0.411	**1.84 [1.45–2.34]**	**< 0.001**
AUDIT-C (ref: low risk)				
Hazardous use	1.10 [0.92–1.31]	0.289	**1.39 [1.07–1.80]**	**0.013**
Harmful use/possible dependence	1.10 [0.87–1.39]	0.436	**3.17 [2.33–4.30]**	**< 0.001**
AUDIT-C (ref: low risk)				
Higher risk	**1.37 [1.17–1.60]**	**< 0.001**	0.84 [0.66–1.07]	0.152

a Adjusted for other demographic factors. Alcohol measures were adjusted for all demographic factors (sex, age, occupational category, and region).

b Ile-de-France (the Parisian region).

Bold: statistically significant. OR, Odds ratio. CI, Confidence intervals.

Regarding alcohol-related measures, individuals who were aware of the campaign (compared to those who were not) were more likely to self-evaluate their drinking as “at-risk” (20% vs. 13%, aOR = 1.65) and to report high-risk consumption based on AUDIT-C (25% vs. 19%, aOR = 1.37). There was no evidence for significant associations between awareness and drinking-related concerns, AUDIT severity or AUD criteria (Supplementary Table S1). Drinking-related concerns, AUDIT scores, and AUDIT-C scores were further described and analyzed as continuous variables, with higher scores observed among individuals aware of the campaign, although effect sizes were small (Supplementary Table S2).

### 3.3 Profile of participants in Dry January

**Figure 2:**
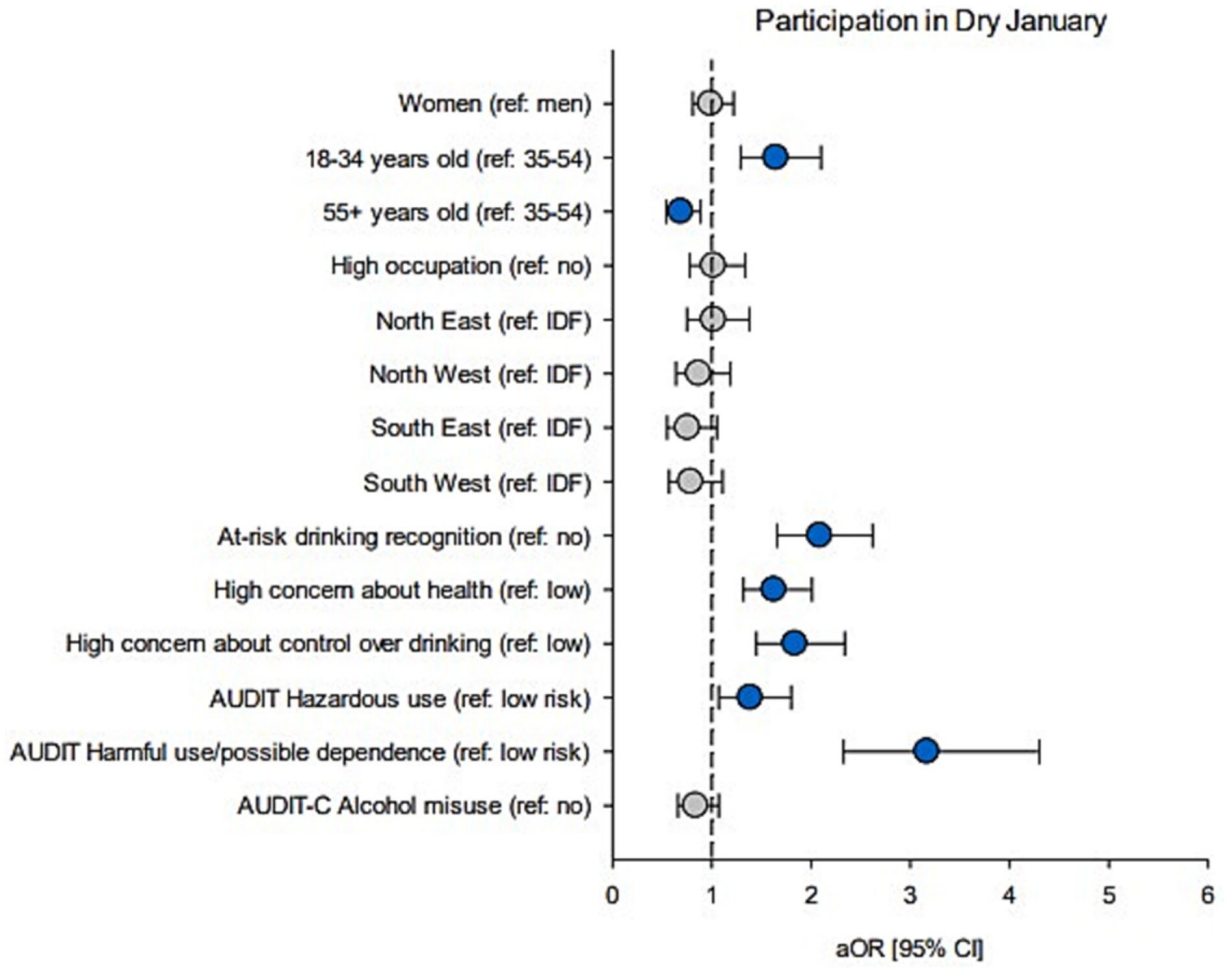
Associations between demographics and alcohol measures, and participation in Dry January. Forest plot shows adjusted OR (circles) and 95% CI (bars); adjusted for demographics. Blue circles: significant estimates (p < 0.05).

Results are reported in Table 1 (demographics), Table 2 (drinking characteristics), and Table 3 (aOR and 95% CI). Figure 2 also reports aOR and 95% CI. Among past-year drinkers who were aware of the Dry January campaign, participation rates did not differ by gender (19% in men vs. 21% in women), occupational category (22% vs. 20%), or region (ranging from 17% in South East to 23% in North East). However, there was evidence for differing participation rates across age groups, with lower participation as age increased (29% among 18–34-year-olds, 20% in 35–54-year-olds, and 15% among those aged 55+). Compared to the 35–54-year-old group, being 18–34 was associated with a higher likelihood of taking part in Dry January (aOR = 1.65), while being 55+ was associated with lower odds of participation (aOR = 0.69).

Regarding drinking profiles, participants in Dry January (compared to non-participants) were more likely to self-identify their drinking as “at-risk” (32% vs. 17%, aOR = 2.09), to be concerned about the health-related effects of alcohol (41% vs. 29%, aOR = 1.63), to be concerned about their control (or lack thereof) over drinking (28% vs. 15%, aOR = 1.84), and to report hazardous use (22% vs. 18%, aOR = 1.39) or harmful use/possible dependence (19% vs. 7%, aOR = 3.17). Accordingly, they were also more likely to meet AUD criteria (Supplementary Table S1). However, there was no evidence for an association between high-risk consumption, as per AUDIT-C, and participation (24% in participants vs. 25% in non-participants). Drinking-related concerns, AUDIT scores, and AUDIT-C scores were further described and analyzed as continuous variables. Drinking-related concerns and AUDIT scores were higher among participants in Dry January. However, they had lower AUDIT-C scores, although the difference was small (Supplementary Table S3). A closer inspection of AUDIT-C data showed that, while participants in Dry January reported drinking less frequently than non-participants (item 1), they were more likely to consume larger amount (item 2) and to report a higher frequency of heavy episodic drinking (HED; item 3). However, these latter outcomes were no longer significant after controlling for demographic factors (see Supplementary Table S4).

#### 3.3.1 Profile of participants in Dry January, according to their initial goal (reduction vs. abstinence)

Results are reported in Table 1 (demographics), Table 4 (drinking characteristics) and Table 5 (aOR and 95% CI). Among participants in Dry January, 95 (19%) indicated they were aiming for reduction rather than abstinence. The proportion of participants aiming for reduction did not differ significantly by gender (21% in men vs. 17% in women), age group (22% among 18–34-year-olds, 18% among 35–54-year-olds, and 17% among people aged 55+), occupational status (25% vs. 18%) or region (ranging from 17 to 20%).

**Table 4:** Drinking characteristics according to Dry January goal and registration status.

	Participants *n* = 497	Abstinence *n* = 402	Reduction *n* = 95	Non-registrants *n* = 395	Registrants *n* = 102
At-risk drinking recognition, n (%)		**χ**^**2**^_**(1)**_ **= 36.908 (*p* < 0.001)**		**χ**^**2**^_**(1)**_ **= 89.09 (*p* < 0.001)**	
	158 (32%)	103 (26%)	55 (58%)	86 (22%)	72 (71%)
High concern about health, n (%)		**χ**^**2**^_**(1)**_ **= 4.755 (*p* = 0.029)**		**χ**^**2**^_**(1)**_ **= 8.045 (*p* = 0.005)**	
	202 (41%)	154 (38%)	48 (51%)	148 (37%)	54 (53%)
High concern about (lack of) control, n (%)		**χ**^**2**^_**(1)**_ **= 12.436 (*p* < 0.001)**		**χ**^**2**^_**(1)**_ **= 58.93 (*p* < 0.001)**	
	137 (28%)	97 (24%)	40 (42%)	78 (20%)	59 (58%)
AUDIT, n (%)		**χ**^**2**^_**(2)**_ **= 36.134 (*p* < 0.001)**		**χ**^**2**^_**(2)**_ **= 118.54 (*p* < 0.001)**	
Low risk	291 (59%)	261 (65%)	30 (32%)	273 (69%)	18 (18%)
Hazardous use	110 (22%)	78 (19%)	32 (34%)	81 (21%)	29 (28%)
Harmful use/possible dependence	96 (19%)	63 (16%)	33 (35%)	41 (10%)	55 (54%)
AUDIT-C: higher risk, n (%)		**χ**^**2**^_**(1)**_ **= 17.146 (*p* < 0.001)**		**χ**^**2**^_**(1)**_ **= 21.545 (*p* < 0.001)**	
	118 (24%)	80 (20%)	38 (40%)	76 (19%)	42 (41%)
Registration, n (%)		**χ**^**2**^_**(1)**_ **= 8.801 (*p* = 0.003)**			
	102 (21%)	72 (18%)	30 (32%)	—	—
Goal: reduction, n (%)				**χ**^**2**^_**(1)**_ **= 8.801 (*p* = 0.003)**	
	95 (19%)	—	—	65 (16%)	30 (29%)

Bold: statistically significant.

**Table 5:** Associations between demographic and alcohol measures, and a goal of reduction and registration.

	Reduction		Registration	
	OR [95% CI]^a^	*p*-value	OR [95% CI]^a^	*p*-value
Gender (ref: men)				
Women	0.76 [0.48–1.22]	0.252	1.02 [0.64–1.63]	0.941
Age (ref: 35–54)				
18–34	1.39 [0.80–2.40]	0.242	**1.83 [1.08–3.10]**	**0.026**
55+	0.96 [0.53–1.74]	0.886	0.66 [0.35–1.25]	0.204
High occupational status (ref: no)	1.64 [0.92–2.93]	0.093	0.72 [0.38–1.36]	0.314
Region (ref: IDF^b^)				
North East	1.31 [0.66–2.61]	0.436	**0.41 [0.21–0.78]**	**0.007**
North West	0.96 [0.46–1.99]	0.905	**0.28 [0.13–0.58]**	**< 0.001**
South East	1.14 [0.54–2.40]	0.738	0.69 [0.35–1.33]	0.266
South West	1.09 [0.52–2.29]	0.826	**0.33 [0.15–0.70]**	**0.004**
At-risk drinking recognition (ref: no)	**3.96 [2.44–6.44]**	**< 0.001**	**8.74 [5.18–14.6]**	**< 0.001**
High concern about health (ref: low)	1.54 [0.97–2.44]	0.067	**1.69 [1.07–2.68]**	**0.026**
High concern about control (ref: low)	**2.13 [1.31–3.47]**	**0.002**	**5.14 [3.15–8.40]**	**< 0.001**
AUDIT (ref: low risk)				
Hazardous use	**4.00 [2.19–7.32]**	**< 0.001**	**5.73 [2.89–11.4]**	**< 0.001**
Harmful use/possible dependence	**5.26 [2.81–9.84]**	**< 0.001**	**21.0 [10.5–41.7]**	**< 0.001**
AUDIT-C (ref: low risk)				
Higher risk	**2.56 [1.56–4.21]**	**< 0.001**	**2.65 [1.62–4.33]**	**< 0.001**

a Adjusted for other demographics. Alcohol measures were adjusted for all demographic factors (sex, age, occupational category, and region).

b Ile-de-France (the Parisian region).

Bold: statistically significant. OR, Odds ratio. CI, Confidence intervals.

In contrast to demographics, there was evidence for differences in drinking characteristics according to the Dry January goal. Participants aiming for reduction (compared to those aiming for abstinence) were more likely to recognize their drinking as at-risk (58% vs. 26%, aOR = 3.96), to be concerned about lack of control over their drinking (42% vs. 24%, aOR = 2.13), to report AUDIT-C-based alcohol misuse (40% vs. 20%, aOR = 2.56), to report hazardous use (34% vs. 19%, aOR = 4.00) and harmful use/possible dependence (35% vs. 16%, aOR = 5.26).They were also more likely to meet AUD criteria (Supplementary Table S5). Drinking-related concerns, AUDIT scores, and AUDIT-C scores were further described and analyzed as continuous variables, showing higher scores among participants aiming for reduction compared to those aiming for abstinence (Supplementary Table S6). Additionally, participants aiming for reduction were more likely to be registrants (32% vs. 18%, aOR = 2.15).

#### 3.3.2 Profile of participants in Dry January, according to their registration status

Results are reported in Table 1 (demographics), Table 4 (drinking characteristics) and Table 5 (aOR and 95% CI). Among the participants in Dry January, 102 (21%) registered to receive emails and supportive messages. Registration rates did not differ by gender (21% of men vs. 20% of women) or occupational status (18% vs. 21%). However, the proportion of participants who registered decreased with age (30% among 18–34-year-olds, 18% in 35–54-year-olds, and 13% among people aged 55+) and varied significantly across regions (from 13% in North West to 33% in the Parisian region). Logistic regression analysis showed that, except for the South East, participants from all other regions were less likely to register compared to those living in the Parisian region.

Registrants exhibited a different drinking profile than non-registrants. Registrants were more likely to recognize their drinking as at-risk (71% vs. 22%, aOR = 8.74), to be concerned about health effects of alcohol (53% vs. 37%, aOR = 1.69), to be concerned about control over their drinking (58% vs. 20%, aOR = 5.14), to report AUDIT-C-based alcohol misuse (41% vs. 19%, aOR = 2.65), hazardous use (28% vs. 21%, aOR = 5.73), and harmful use/possible dependence (54% vs. 10%, aOR = 21.0). Additionally, registrants were more likely to meet AUD criteria (Supplementary Table S5). Drinking-related concerns, AUDIT scores, and AUDIT-C scores were further described and analyzed as continuous variables, with higher scores observed among registrants compared to non-registrants (Supplementary Table S7).

## 4 Discussion

Among alcohol users, 61% were aware of the “Dry January” campaign, with higher awareness among those with high occupational status, and lower awareness in Eastern regions. Those aware of the campaign were more likely to assess their drinking as “at risk” and report high-risk consumption. Of the alcohol users aware of the campaign, 20% were participants (12% of all users). Participation decreased with age. Compared to non-participants, Dry January participants were more likely to self-identify as at-risk drinkers, express concerns about alcohol’s health effects and control over drinking, and report hazardous use and alcohol-related harms. Among participants, aiming for reduction (rather than abstinence) and campaign registration were associated with worsened alcohol-related outcomes.

### 4.1 Awareness of the campaign

Four years after the first Dry January campaign in France (“le Défi de Janvier”), 61% of past-year alcohol users were aware of the initiative, a similar proportion to that observed during the first campaign. In fact, a recent report from *Santé Publique France* indicated that in 2020 (the first campaign) and 2021, 63 and 53% of adults (aged 18–75) respectively, had heard of a “campaign promoting abstaining from alcohol in January” (without explicitly using the term “Dry January”) (33). According to the authors, the sharp decline in 2021 was likely due to greater press and media coverage during the first edition, along with the 2021 campaign occurring during the COVID-19 pandemic, which may have limited its visibility. Similar surveys of population-representative samples of drinkers in England showed that 64 and 78% were aware of the campaign in 2015 and 2016, respectively (i.e., 2 and 3 years after the first campaign) (19). Notably, in 2015, Public Health England supported the promotion of Dry January, leading to substantial investment in radio, press, and social media advertising. In contrast, Dry January in France, has been primarily promoted by health charities and has not received government support. The French government’s withdrawal from the initial launch deprived the campaign of necessary funding for advertising, including a dedicated website and a smartphone application that contribute to social contagion and diffusion, which have been shown as crucial for improving awareness and participation in the campaign (19). Despite this, our results indicate that Dry January is still relatively well-recognized by the French adult population. However, the findings also highlight the need for enhanced mass communication strategies to maximize the campaign’s reach, particularly given the variations in awareness across occupational statuses and regions. We did not initially formulate specific hypotheses regarding the demographic factors influencing awareness of Dry January, primarily due to a lack of published research on the topic. However, the recent report from *Santé Publique France*, based on 2021 data, offers valuable insights (33). The report indicated that younger individuals were less likely to be aware of Dry January, while higher socio-economic status—as measured by education level and income—was linked to greater awareness. These latter findings are consistent with our results, where higher occupational status was similarly associated with a higher likelihood of awareness. Better access to information, a stronger focus on health-related behaviors, and social networks that promote health trends may explain why awareness of Dry January is more prevalent among these individuals. However, we did not find a significant association between age and awareness. This discrepancy might suggest that awareness of the campaign has expanded over time, with younger individuals now more familiar with it than they were during the initial campaigns. Regarding regional differences, one possible explanation could be varying levels of media coverage, and local health promotion efforts across regions. Additionally, given the documented regional disparities in alcohol consumption patterns across France (5), regional cultural norms surrounding alcohol consumption may influence the visibility and relevance of Dry January in different areas.

### 4.2 Participation in the campaign

Among past-year drinkers aware of the campaign, 20% reported participating (12% of all drinkers), a distinct increase compared to 2020 and 2021, where 8 and 9% of past-year alcohol drinkers who were aware of the campaign reported a change in their drinking in relation to the initiative (4.5% of all past-year drinkers in both 2020 and 2021) (33). These findings are encouraging and align with studies conducted in England that show a steady increase in Dry January participation over the years (19, 23, 30). Similarly, a market research poll reported that 25% of 1,506 U.S. adults participated in Dry January 2024 (34), compared to 16% in 2023 (35). In the 2024 survey, 35% of those aged 21–24 and 31% of those aged 25–34 participated in the campaign. In contrast, a recent study of U.S. emerging adults (aged 18–29) found that, in 2021–2022, only 7% had participated in a TAAC in the past year (36).

### 4.3 Profile of participants

Participation in Dry January was comparable across gender and occupational status, but decreased with age. While this contrasts with international cohort studies suggesting that women and individuals with higher socio-economic status are more likely to participate in TAACs (6, 25), our findings align with surveys conducted on population-representative samples (30, 33). This suggests that the over-representation of these demographic categories in cohort studies may be due to their higher likelihood of participating in mail or web surveys (37, 38). Our results are, however, consistent with surveys reporting higher participation rates among younger individuals (33, 34). Interestingly, a French qualitative study on TAAC participants found that younger people tend to have a heightened perception of some health risks associated with alcohol consumption (39). In the same study, younger individuals were more likely to report that heavy episodic drinking and its negative consequences (e.g., vomiting, blackout, and degraded self-image) were difficult to tolerate, leading them to question their relationship with alcohol and its effects.

Consistent with previous findings (6, 36), this study shows that Dry January participants were more likely to self-identify their drinking as “at-risk,” express concerns about health-related effects of alcohol and control over their drinking, and report hazardous use or harmful use/possible dependence. Accordingly, participants were also more likely to endorse DSM-5 AUD criteria. However, caution is needed when interpreting these results clinically due to the self-reported nature of the study. Interestingly, we found no evidence for an association between participation and high-risk consumption as measured by the AUDIT-C (defined as score ≥ 5). Further analysis revealed that although participants reported drinking less frequently than non-participants, they were more likely to consume larger amounts of alcohol and report higher frequency of HED. However, these latter outcomes were no longer significant after controlling for demographic factors. Taken together, these findings underscore the importance of distinguishing between the frequency of alcohol consumption and the experience of negative consequences or harms associated with drinking, as well as the feeling of losing control over alcohol use. One possible hypothesis is that participants are already engaged in behavioral changes regarding their alcohol use, specifically by drinking less frequently while still engaging in HED. This is compatible with our data showing that Dry January participants had higher rates of at-risk drinking recognition and reported more negative consequences or harmful effects related to alcohol consumption, as reflected by (total) AUDIT scores and DSM-5 criteria endorsement.

Overall, these findings confirm that TAACs such as Dry January may primarily attract individuals who are at higher risk of alcohol-related harm. They may recognize the negative impact alcohol has on their health and well-being, leading them to seek ways to regain control over their drinking habits. It is worth noting that our results differ from de Visser and Piper (2020) who reported that Dry January participants had higher AUDIT-C scores than non-participants (21). However, in their study, 85% of Dry January participants were registrants, compared to 21% in the current study. Our results indicate that all drinking measures were higher (or worsened) among registrants compared to non-registrants. This suggests that registrants may not be representative of the broader TAAC participant population and likely constitute a subgroup at higher risk of harmful consumption, or at least perceive themselves as such, thus being more inclined to seek support. Consistently, registrants were more likely to aim for reduction rather than abstinence. While TAACs typically encourage drinkers to abstain from alcohol for a set period, anecdotal evidence suggests that some individuals participate with the goal of reducing their alcohol intake rather than abstaining completely. Recently, Thienpondt et al. (2024) reported that 91% of participants in the *Tournée Minérale* campaign—a TAAC launched in Belgium in 2017—aimed to totally abstain from alcohol, while 9% aimed to reduce their alcohol consumption (25). Similarly, in our study, most Dry January participants aimed for abstinence (81%) while 19% aimed to reduce their drinking (we are not aware of other published studies assessing this outcome). Although no demographic factors were significantly associated with this outcome, participants aiming for reduction were more likely to report high-risk consumption, alcohol-related harms and concerns. In this context, reducing alcohol consumption may be seen as a more achievable goal than full abstinence. These findings highlight the need for future studies to assess and account for the initial goals of TAAC participants.

### 4.4 Alcohol consumption in France

In this sample of 5,000 respondents, 82% were past-year alcohol users, a slightly lower prevalence than those from the Health Barometer surveys of 2017 and 2021, which found 86.5 and 85% of past-year drinkers in representative samples of the adult population, respectively (4). In this study, 18% reported hazardous use (AUDIT scores from 8 to 15), and 9% reported harmful use or possible dependence (AUDIT score > 15). To further compare our data with the most recent national survey, supplementary descriptive analyses showed that 10% of past-year drinkers reported consuming alcohol “4 times or more per week” (AUDIT item 1), while 12 and 4% indicated monthly and weekly HED, respectively (i.e., ≥ 6 standard alcoholic drinks in one occasion, AUDIT item 3; see Supplementary Table S8). Overall, these results are consistent with the 2021 survey, which reported 8% of daily alcohol consumption and 16.5 and 5% of monthly and weekly HED, respectively (4). However, prevalence rates of AUD, based on DSM-5 criteria, were notably high in our sample: 18% (mild), 7% (moderate), and 11% (severe). These findings raise questions about the validity of using self-reported DSM diagnostic criteria in epidemiological studies (40–42). Investigating the validity of self-reported DSM-5 severity levels for AUD in relation to the AUDIT is beyond the scope of this study and will be the subject of future analysis.

### 4.5 Limitations

This study has several limitations. First, the data were self-reported, and responses may be subject to recall bias such as inaccuracies in remembering alcohol consumption. Second, the recruitment method may have introduced selection bias, as the sample was drawn from a panel of individuals who had expressed interest in participating in research surveys, which may not be representative of the wider population, including vulnerable groups. Third, the cross-sectional nature of the study prevents us from making causal inferences about the observed associations. Fourth, the study was conducted from 8^th^ to 17^th^ of January, and it is possible that some individuals who consumed alcohol early in the month no longer considered themselves as “participants” in Dry January. Fifth, the demographic variables were somewhat limited and did not include factors such as marital status or education. Additionally, contextual information, such as prior participation or motivations for participating, were not investigated. Finally, the question evaluating awareness referred specifically to the name “Dry January” without explaining the concept as “an alcohol-free month.” Awareness rates may therefore be underestimated, as some people might have recognized the concept more readily than the name. However, it is unlikely that individuals participating in the campaign would be unfamiliar with its name.

## 5 Conclusion

This study demonstrated that a significant proportion (61%) of alcohol users in France are aware of the Dry January campaign. Participation in Dry January was encouraging with 12% of alcohol users taking part, representing a distinct increase from the first campaigns. Importantly, this study confirmed that participants were more likely to report hazardous use, negative consequences associated with their alcohol consumption, and concerns about their drinking, especially among those who registered to receive supportive messages and tips. In other words, Dry January appears to fulfill one of its key objectives by attracting individuals at higher risk of alcohol-related harms, i.e., those who are most likely to benefit from such a campaign. As part of the JANOVER research project, an ongoing prospective cohort study will provide valuable insights into the benefits associated with TAAC in France.

## Data Availability

The raw data supporting the conclusions of this article will be made available by the authors, without undue reservation.

## Footnotes

^1^ https://jasp-stats.org/
