# Supplementary material for: Prevalence and characteristics of participants in Dry January 2024: findings from a general population survey in France
Source: Front Public Health. 2024 Dec 2;12:1466739. doi: 10.3389/fpubh.2024.1466739 (PMC11647028; doi:10.3389/fpubh.2024.1466739)
Supplement: Supplementary file 1 [file Table_1.docx]

Supplementary Material

Prevalence and Characteristics of Participants in *Dry January* 2024: Findings from a General Population Survey in France

**Table S1.** AUD criteria according to awareness of, and participation in Dry January.

|  |  | Among past-year drinkers | | Among people aware of the campaign | |
| --- | --- | --- | --- | --- | --- |
|  | Past-year drinkers  n = 4,075 | Unaware  n = 1,607 | Aware  n = 2,468 | Non-participants  n = 1,971 | Participants  n = 497 |
| DSM-5 AUD, n (%) |  | **χ²_(3)_ = 9.320 (*p* = .025)** | | **χ²_(3)_ = 143.53 (*p* < .001)** | |
| None | 2,634 (65%) | 1,084 (67%) | 1,550 (63%) | 1,319 (67%) | 231 (46%) |
| Mild | 735 (18%) | 269 (17%) | 466 (19%) | 363 (18%) | 103 (21%) |
| Moderate | 274 (7%) | 100 (6%) | 174 (7%) | 139 (7%) | 35 (7%) |
| Severe | 432 (11%) | 154 (10%) | 278 (11%) | 150 (8%) | 128 (26%) |

|  | Awareness | | Participation | |
| --- | --- | --- | --- | --- |
|  | OR [95% CI]^a^ | *p*-value | OR [95% CI]^a^ | *p*-value |
| DSM-5 AUD (ref: no AUD) |  |  |  |  |
| Mild | 1.17 [0.99 – 1.40] | .069 | **1.55 [1.18 – 2.02]** | **.001** |
| Moderate | 1.16 [0.89 – 1.51] | .289 | 1.35 [0.90 – 2.02] | .149 |
| Severe | 1.20 [0.96 – 1.50] | .105 | **4.30 [3.22 – 5.75]** | **< .001** |

^a^ Adjusted for all demographic factors (sex, age, occupational category, and region).

Bold: statistically significant.

OR = Odds ratio. CI = Confidence intervals.

**Table S2.** Drinking-related concerns, AUDIT scores, and AUDIT-C scores, according to awareness of Dry January. Due to non-normality, comparisons between groups were performed with Mann-Whitney tests. Effect size is given by rank biserial correlation (*r_rb_*). Mean and SD are also reported for the sake of description.

|  | Past-year drinkers  n = 4,075 | Unaware of Dry January  n = 1,607 | Aware of Dry January  n = 2,468 | *p*-value | Effect size |
| --- | --- | --- | --- | --- | --- |
| Concern about health |  |  |  |  |  |
| Median (IQR) | 3 (1 – 7) | 3 (0 – 7) | 3 (1 – 7) | **.035** | ***r_rb_* = .04** |
| Mean (SD) | 3.8 (3.3) | 3.7 (3.4) | 3.9 (3.2) |  |  |
| Concern about (lack of) control |  |  |  |  |  |
| Median (IQR) | 2 (0 – 5) | 2 (0 – 5) | 1 (0 – 5) | .522 | *r_rb_* = .01 |
| Mean (SD) | 2.7 (3.0) | 2.8 (3.1) | 2.7 (3.0) |  |  |
| AUDIT score |  |  |  |  |  |
| Median (IQR) | 4 (2 – 8) | 4 (2 – 8) | 4 (2 – 8) | **.006** | ***r_rb_* = .08** |
| Mean (SD) | 6.2 (5.9) | 5.9 (5.8) | 6.4 (5.9) |  |  |
| AUDIT-C score |  |  |  |  |  |
| Median (IQR) | 3 (2 – 4) | 3 (2 – 4) | 3 (2 – 5) | **< .001** | ***r_rb_* = .11** |
| Mean (SD) | 3.3 (1.9) | 3.1 (1.8) | 3.5 (2.0) |  |  |

IQR: interquartile range. SD: standard deviation.

**Table S3.** Drinking-related concerns, AUDIT scores, and AUDIT-C scores, according to participation in Dry January. Due to non-normality, comparisons between groups were performed with Mann-Whitney tests. Effect size is given by rank biserial correlation (*r_rb_*). Mean and SD are also reported for the sake of description.

|  | Aware of Dry January  n = 2,468 | Non-participants  n = 1,971 | Participants  n = 497 | *p*-value | Effect size |
| --- | --- | --- | --- | --- | --- |
| Concern about health |  |  |  |  |  |
| Median (IQR) | 3 (1 – 7) | 3 (1 – 6) | 5 (1 – 8) | **< .001** | ***r_rb_* = .17** |
| Mean (SD) | 3.9 (3.2) | 3.7 (3.2) | 4.7 (3.5) |  |  |
| Concern about (lack of) control |  |  |  |  |  |
| Median (IQR) | 1 (0 – 5) | 1 (0 – 4) | 2 (0 – 6) | **< .001** | ***r_rb_* = .18** |
| Mean (SD) | 2.7 (3.0) | 2.4 (2.8) | 3.5 (3.4) |  |  |
| AUDIT score |  |  |  |  |  |
| Median (IQR) | 4 (2 – 8) | 4 (2 – 8) | 5 (2 – 13) | **< .001** | ***r_rb_* = .13** |
| Mean (SD) | 6.4 (5.9) | 5.9 (5.3) | 8.4 (7.7) |  |  |
| AUDIT-C score |  |  |  |  |  |
| Median (IQR) | 3 (2 – 5) | 3 (2 – 5) | 3 (2 – 4) | **.020** | ***r_rb_* = .07** |
| Mean (SD) | 3.5 (2.0) | 3.5 (2.0) | 3.3 (2.1) |  |  |

IQR: interquartile range. SD: standard deviation.

**Table S4.** Responses to AUDIT-C items according to participation in Dry January.

|  | Non-participants  n = 1,971 | Participants  n = 497 |
| --- | --- | --- |
| 1. How often do you have a drink containing alcohol? | | |
|  | **χ²_(3)_ = 76.629 (*p* < .001)** | |
| Monthly or less | 435 (22%) | 191 (38%) |
| 2-4 times a month | 749 (38%) | 190 (38%) |
| 2-3 times a week | 528 (27%) | 92 (19%) |
| 4 or more times a week | 259 (13%) | 24 (5%) |
| 2. How many standard drinks containing alcohol do you have on a typical day when drinking? | | |
|  | **χ²_(4)_ = 11.007 (*p* = .026)** | |
| 1 or 2 | 1,346 (68%) | 305 (61%) |
| 3 or 4 | 477 (24%) | 139 (28%) |
| 5 or 6 | 113 (6%) | 41 (8%) |
| 7 to 9 | 21 (1%) | 9 (2%) |
| 10 or more | 14 (1%) | 3 (1%) |
| 3. How often do you have six or more drinks on one occasion? | | |
|  | **χ²_(4)_ = 15.372 (*p* = .004)** | |
| Never | 924 (47%) | 209 (42%) |
| Less than monthly | 707 (36%) | 166 (33%) |
| Monthly | 239 (12%) | 84 (17%) |
| Weekly | 74 (4%) | 31 (6%) |
| Daily or almost daily | 27 (1%) | 7 (1%) |
| **Logistic regression^a^** | **Participation** | |
|  | **OR [95% CI]^a^** | ***p*-value** |
| Item 1 (ref: monthly or less) |  |  |
| 2-4 times a month | **0.58 [0.46 – 0.74]** | **< .001** |
| 2-3 times a week | **0.40 [0.30 – 0.53]** | **< .001** |
| 4 or more times a week | **0.25 [0.15 – 0.39]** | **< .001** |
| Item 2 (ref: 1 or 2) |  |  |
| 3 or 4 | 1.20 [0.95 – 1.51] | .133 |
| 5 or 6 | 1.37 [0.93 – 2.02] | .112 |
| 7 to 9 | 1.57 [0.70 – 3.53] | .270 |
| 10 or more | 0.75 [0.21 – 2.69] | .662 |
| Item 3 (ref: never) |  |  |
| Less than monthly | 0.88 [0.70 – 1.12] | .309 |
| Monthly | 1.23 [0.90 – 1.68] | .195 |
| Weekly | 1.41 [0.88 – 2.24] | .151 |
| Daily or almost daily | 1.06 [0.45 – 2.50] | .896 |

^a^ adjusted for demographic factors (sex, age, occupational category, and region).

**Table S5.** AUD criteria according to Dry January goal and registration status.

|  | Participants  n = 497 | Abstinence  n = 402 | Reduction  n = 95 | Non-registrants  n = 395 | Registrants  n = 102 |
| --- | --- | --- | --- | --- | --- |
| DSM-5 AUD, n (%) |  | **χ²_(3)_ = 36.814 (*p* < .001)** | | **χ²_(3)_ = 142.79 (*p* < .001)** | |
| None | 231 (46%) | 209 (53%) | 22 (23%) | 219 (55%) | 12 (11%) |
| Mild | 103 (21%) | 85 (21%) | 18 (19%) | 94 (24%) | 9 (9%) |
| Moderate | 35 (7%) | 22 (5%) | 13 (14%) | 26 (7%) | 9 (9%) |
| Severe | 128 (26%) | 86 (21%) | 42 (44%) | 56 (14%) | 72 (71%) |

|  | Reduction | | Registration | |
| --- | --- | --- | --- | --- |
|  | OR [95% CI]^a^ | *p*-value | OR [95% CI]^a^ | *p*-value |
| DSM-5 AUD (ref: no AUD) |  |  |  |  |
| Mild | **2.20 [1.10 – 4.42]** | **.027** | 1.68 [0.67 – 4.22] | .273 |
| Moderate | **5.99 [2.59 – 13.8]** | **< .001** | **6.21 [2.31 – 16.7]** | **< .001** |
| Severe | **5.17 [2.75 – 9.73]** | **< .001** | **25.6 [12.1 – 54.0]** | **< .001** |

^a^ Adjusted for all demographic factors (sex, age, occupational category, and region).

Bold: statistically significant.

OR = Odds ratio. CI = Confidence intervals.

**Table S6.** Drinking-related concerns, AUDIT scores, and AUDIT-C scores, according to Dry January goal. Due to non-normality, comparisons between groups were performed with Mann-Whitney tests. Effect size is given by rank biserial correlation (*r_rb_*). Mean and SD are also reported for the sake of description.

|  | Participants  n = 497 | Abstinence  n = 402 | Reduction  n = 95 | *p*-value | Effect size |
| --- | --- | --- | --- | --- | --- |
| Concern about health |  |  |  |  |  |
| Median (IQR) | 5 (1 – 8) | 4 (1 – 8) | 6 (4 – 8) | **< .001** | ***r_rb_* = .24** |
| Mean (SD) | 4.7 (3.5) | 4.5 (3.6) | 5.9 (2.7) |  |  |
| Concern about (lack of) control |  |  |  |  |  |
| Median (IQR) | 2 (0 – 6) | 2 (0 – 5) | 5 (2 – 8) | **< .001** | ***r_rb_* = .35** |
| Mean (SD) | 3.5 (3.4) | 3.2 (3.4) | 5.0 (3.0) |  |  |
| AUDIT score |  |  |  |  |  |
| Median (IQR) | 5 (2 – 13) | 4 (2 – 11.8) | 11 (5 – 18) | **< .001** | ***r_rb_* = .41** |
| Mean (SD) | 8.4 (7.7) | 7.3 (7.0) | 12.7 (8.9) |  |  |
| AUDIT-C score |  |  |  |  |  |
| Median (IQR) | 3 (2 – 4) | 3 (2 – 4) | 4 (3 – 6) | **< .001** | ***r_rb_* = .36** |
| Mean (SD) | 3.3 (2.1) | 3.1 (1.9) | 4.4 (2.3) |  |  |

IQR: interquartile range. SD: standard deviation.

**Table S7.** Drinking-related concerns, AUDIT scores, and AUDIT-C scores, according to registration. Due to non-normality, comparisons between groups were performed with Mann-Whitney tests. Effect size is given by rank biserial correlation (*r_rb_*). Mean and SD are also reported for the sake of description.

|  | Participants  n = 497 | Non-registrants  n = 395 | Registrants  n = 102 | *p*-value | Effect size |
| --- | --- | --- | --- | --- | --- |
| Concern about health |  |  |  |  |  |
| Median (IQR) | 5 (1 – 8) | 4 (1 – 8) | 6 (4 – 9) | **< .001** | ***r_rb_* = .33** |
| Mean (SD) | 4.7 (3.5) | 4.3 (3.5) | 6.3 (3.0) |  |  |
| Concern about (lack of) control |  |  |  |  |  |
| Median (IQR) | 2 (0 – 6) | 2 (0 – 5) | 6.5 (4 – 9) | **< .001** | ***r_rb_* = .55** |
| Mean (SD) | 3.5 (3.4) | 2.8 (3.1) | 6.2 (3.1) |  |  |
| AUDIT score |  |  |  |  |  |
| Median (IQR) | 5 (2 – 13) | 4 (2 – 9) | 16 (11.25 – 20.75) | **< .001** | ***r_rb_* = .67** |
| Mean (SD) | 8.4 (7.7) | 6.4 (6.1) | 16.1 (8.3) |  |  |
| AUDIT-C score |  |  |  |  |  |
| Median (IQR) | 3 (2 – 4) | 3 (2 – 4) | 4 (3 – 6) | **< .001** | ***r_rb_* = .45** |
| Mean (SD) | 3.3 (2.1) | 3.0 (1.9) | 4.6 (2.2) |  |  |

IQR: interquartile range. SD: standard deviation.

**Table S8.** Highest consumption frequency and heavy episodic drinking among past-year drinkers based on AUDIT-C.

|  | Men (n=2,050) | Women (n=2,025) | Total (n = 4,075) |
| --- | --- | --- | --- |
| 4 or more times per week (item 1^a^) | 301 (15%) | 106 (5%) | 407 (10%) |
| Monthly heavy episodic drinking (item 3^b^) | 298 (15%) | 209 (10%) | 507 (12%) |
| Weekly heavy episodic drinking (item 3) | 104 (5%) | 58 (3%) | 162 (4%) |

^a^ *How often do you have a drink containing alcohol?*

^b^ *How often do you have six or more drinks on one occasion?*
